# Geographic Accessibility of COVID-19 Test to Treat Sites by Race, Ethnicity, Age, and Rurality

**DOI:** 10.1001/jamanetworkopen.2022.41144

**Published:** 2022-11-09

**Authors:** Rohan Khazanchi, Andrew Strumpf, Utibe R. Essien, Samuel D. Powers, Kathleen A. McManus

**Affiliations:** 1 Department of Internal Medicine, Harvard Medical School, Boston, Massachusetts; 2 Department of Pediatrics, Harvard Medical School, Boston, Massachusetts; 3 Internal Medicine-Pediatrics Residency Program, Brigham and Women’s Hospital, Boston Children’s Hospital, Boston Medical Center, Boston, Massachusetts; 4 Division of Infectious Diseases and International Health, Department of Medicine, University of Virginia School of Medicine, Charlottesville; 5 Center for Health Equity Research and Promotion, Veterans Affairs Pittsburgh Healthcare System, Pittsburgh, Pennsylvania; 6 Center for Pharmaceutical Policy and Prescribing, University of Pittsburgh School of Medicine, Pittsburgh, Pennsylvania; 7 Global Infectious Diseases Institute, University of Virginia, Charlottesville

## Abstract

This cross-sectional study explores geographic disparities in antiviral access by quantifying the accessibility of COVID-19 Test to Treat sites for subpopulations by race, ethnicity, age, and rurality.

## Introduction

Nirmatrelvir-ritonavir and molnupiravir are oral antivirals that reduce risk of hospitalization for people with mild to moderate COVID-19.^1^ Timely access to treatment is a priority because these medications are indicated within 5 days of symptom onset. In March 2022, the Biden Administration announced the Test to Treat initiative to designate one-stop locations where people can receive a COVID-19 test, speak with a clinician, obtain an antiviral prescription, and fill the prescription for free.^2^ However, concerns remain that the Test to Treat program may not be accessible for minoritized and high-risk populations.^3,4,5^ To explore geographic disparities in antiviral access, we quantified the accessibility of Test to Treat sites for subpopulations by race, ethnicity, age, and rurality.

## Methods

We analyzed published geolocations of all COVID-19 Test to Treat sites from HealthData.gov as of May 4, 2022. We calculated drive times from the population center of every census tract to the 10 geographically closest sites and identified the shortest time. We then calculated the national proportion of each demographic subgroup residing within *x* minutes of the nearest site by weighting all tracts by population size, stratifying by tract-level population demographics (rurality, age, race, and ethnicity; obtained from the 2010 US Department of Agriculture Economic Research Service Rural-Urban Commuting Area codes and 2015-2019 US Census American Community Survey), and nationally aggregating weighted drive times by demographic subgroup. We calculated subgroup median drive times with 95% CIs by bootstrap. The study was approved by the University of Virginia institutional review board as research not involving human participants; therefore, no informed consent was required. This study followed the STROBE reporting guideline for cross-sectional studies. We analyzed data using ArcGIS Pro, version 2.8.6 (Esri) and R, version 4.2.0 (R Group for Statistical Computing)

## Results

**Figure 1.**
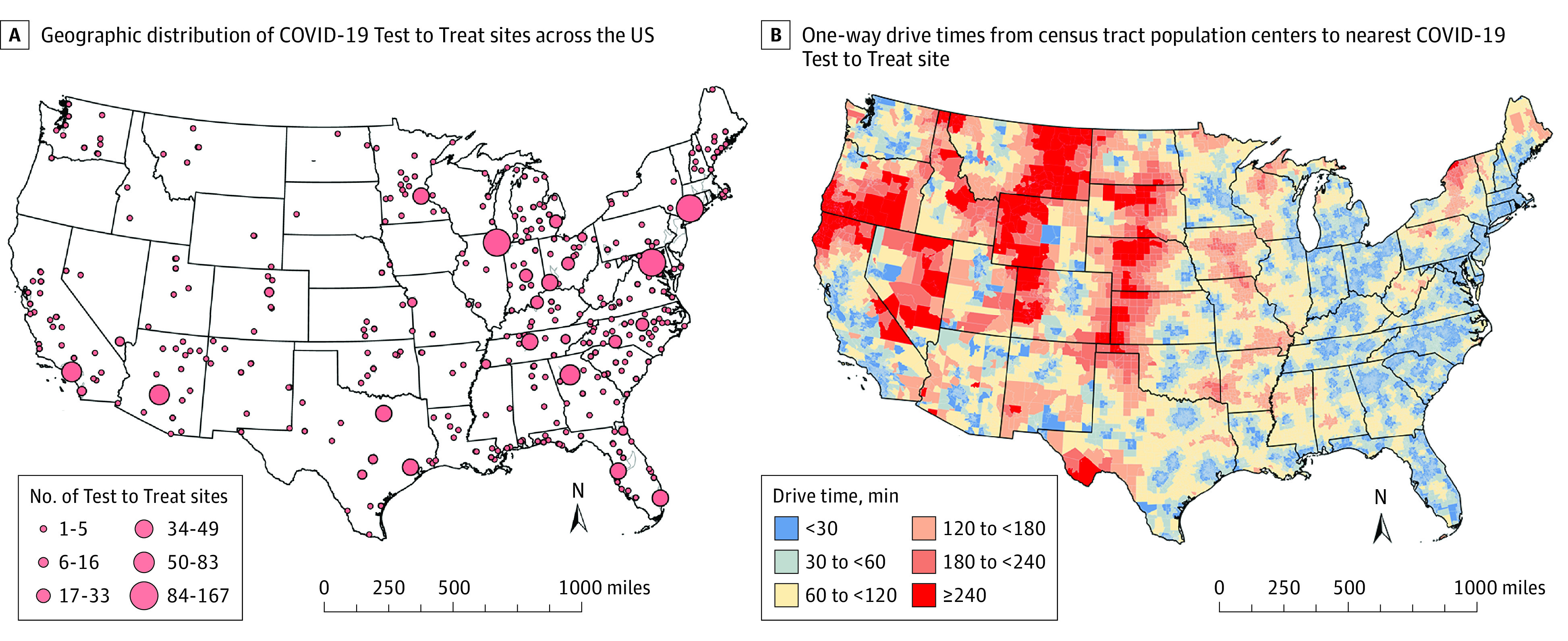
Geographic Distribution of COVID-19 Test to Treat Sites and One-Way Drive Times From Census Tract Population Centers to Nearest COVID-19 Test to Treat Site Across the US Maps were created using ArcGIS Pro, version 2.8.6 (Esri). A, To display the number of Test to Treat sites within an area of geographic proximity, all trial sites were plotted, and those within 25 miles of each other were aggregated into polygons. Graduated circles indicating the number of aggregated sites were plotted at the centroid of each polygon. B, One-way drive time was calculated from the center of population within each census tract to the nearest Test to Treat site.

**Figure 2.**
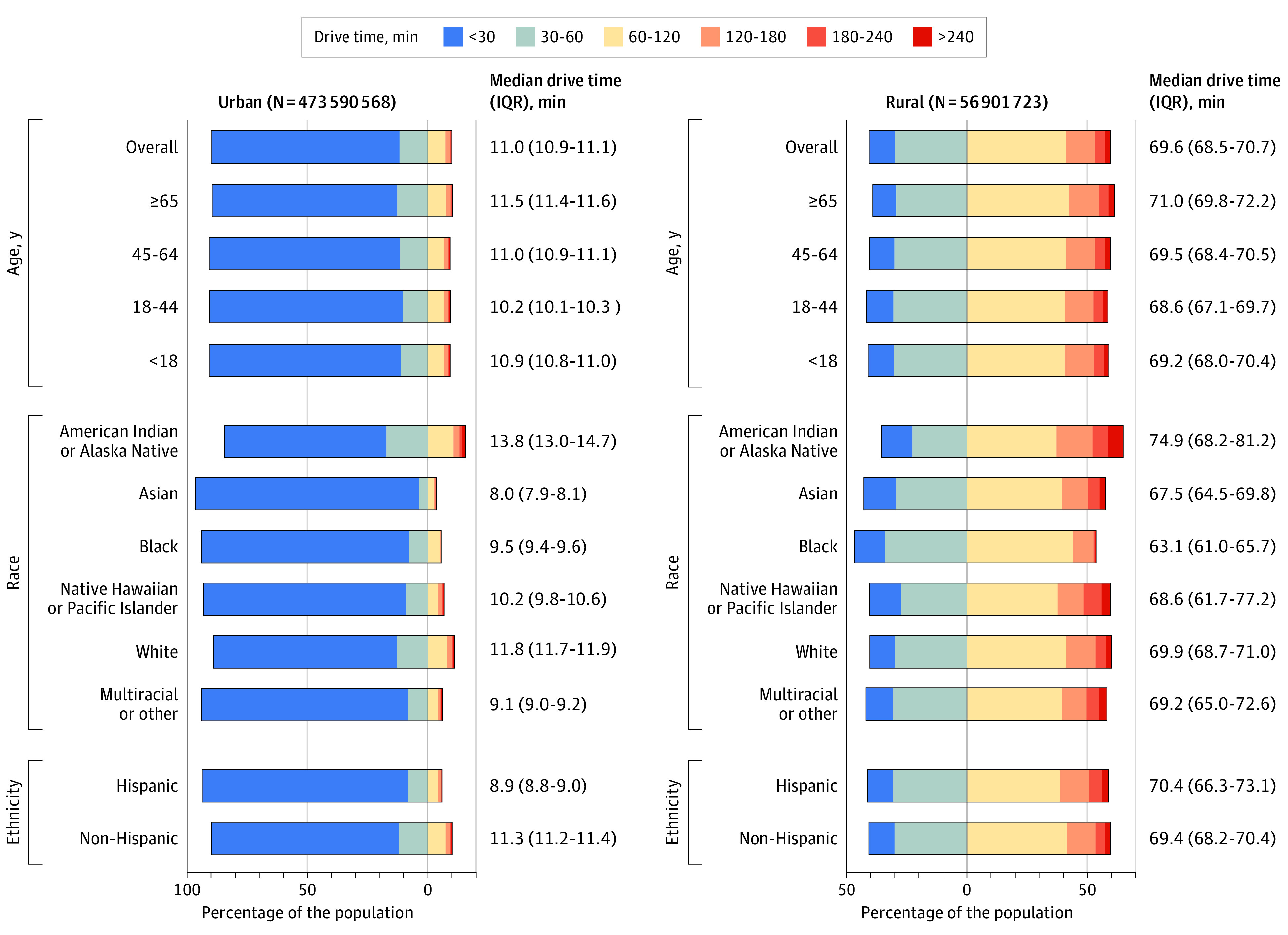
Demographic Differences in One-Way Drive Times to the Nearest COVID-19 Test to Treat Site The percentage of the population with shorter than (ie, left of 0% on the x-axis) or longer than (ie, right of 0% on the x-axis) a 60-minute drive time to the nearest Test to Treat site is shown. For each sociodemographic subgroup, the median and 95% CIs are displayed to the right of the bar.

We identified 2227 unique Test to Treat sites. The sites were concentrated around metropolitan centers, with shorter drive times most noticeable near urban areas (Figure 1). Overall, 15% of the US population, but 59% of rural residents, lived more than 60 minutes from the nearest site. In total, 17% of elderly (aged ≥65 years) individuals, 30% of American Indian or Alaskan Native individuals, 17% of White individuals, 8% of Hispanic individuals, and 8% of Black individuals lived more than 60 minutes away from the nearest site (Figure 2).

Compared with White individuals (median, 13.9 [95% CI, 13.8-14.1] minutes), American Indian or Alaskan Native individuals (median, 28.5 [95% CI, 25.9-31.1] minutes) lived farther from sites, whereas Asian individuals (median, 8.0 [95% CI, 7.9-8.1] minutes), Hispanic individuals (median, 9.2 [95% CI, 9.1-9.4] minutes), and Black individuals (median, 10.0 [95% CI, 9.9-10.1] minutes) lived closer to sites. Rural residents (median, 69.2 [95% CI, 68.5-70.7] minutes) had longer drive times than urban residents (median, 11.0 [95% CI, 10.9-11.1] minutes), consistent across all demographic subgroups (Figure 2). American Indian or Alaskan Native individuals had the longest median drive times among both urban (13.8 [95% CI, 13.0-14.7] minutes) and rural (74.9 [95% CI, 68.2-81.2] minutes) subpopulations.

## Discussion

This cross-sectional study found that approximately 15% of the overall US population, 30% of American Indian or Alaskan Native people, and 59% of the rural population lived more than 60 minutes from the nearest site. Rural populations had a median 58-minute longer drive to the nearest site compared with urban populations. American Indian or Alaskan Native populations had longer drive times even after accounting for rurality, suggesting that they are uniquely isolated from antiviral access despite bearing a disproportionate COVID-19 burden. Expanding inclusion of rural and tribal facilities in the Test to Treat initiative may improve access for these populations.

Asian, Black, and Hispanic populations lived closer to sites, but geographic accessibility alone is insufficient for pharmacoequity. These populations have been less likely to receive outpatient COVID-19 therapeutics than White individuals despite elevated risk of infection and severe disease.^5^ This inequity may be associated with low antiviral dispensing rates in areas with highest social vulnerability.^3,4^ Equitable distribution schemes should ensure that federally qualified health centers, safety-net hospitals, and local pharmacies are well represented in the Test to Treat initiative; allocate resources based on equity metrics and community-identified needs; and prioritize “low-tech, high-touch” outreach that leverages trusted community stakeholders for in-person outreach.^3,6^

Limitations include our use of tract population centers, which assumes that demographic subgroups are not clustered within tracts. Drive time is a widely accepted proxy for geographic accessibility but does not account for unequal transportation access. Interventions are needed to reduce geographic heterogeneity in antiviral availability.
